# Enhanced growth of large-scale nanostructures with metallic ion precipitation in helium plasmas

**DOI:** 10.1038/s41598-017-18476-7

**Published:** 2018-01-08

**Authors:** Shin Kajita, Shota Kawaguchi, Noriyasu Ohno, Naoaki Yoshida

**Affiliations:** 1 Institute of Materials and Systems for Sustainability, Nagoya University, Nagoya, 464-8603 Japan; 2 Graduate School of Engineering, Nagoya University, Nagoya, 464-8603 Japan; 3 Research Institute for Applied Mechanics, Kyushu University, Fukuoka, 816-8580 Japan

## Abstract

Helium plasma irradiation on metal surfaces leads to the formation of metallic fuzzy nanostructures accompanied by the growth of helium bubbles in metals. The mechanism of the growth process, its impact for fusion devices, and potential application have been explored. Here we show enhanced growth of large-scale fuzz by precipitating additional metallic particles during helium plasma irradiation. The growth rate of the fuzzy structures became orders of magnitude greater than conventional fuzz growth; in an hour of irradiation, 1 mm-thick visible tungsten and molybdenum fuzzy fur structures covered a tungsten metal substrate. Additional precipitation of metallic ions breaks the bottleneck diffusion process; moreover, further acceleration in the growth rate could have occurred if the electric sheath shape was influenced by the grown structure and the electric field that formed around the structure started collecting ions.

## Introduction

The interaction between tungsten (W) and helium (He) is an important issue in a fusion environment. It has been investigated for many years, because He atoms are by-products of nuclear fusion reactions^1,2^. The growth of nanostructures called *fuzz* on metallic surfaces due to helium plasma irradiation was initially identified on W surfaces in a demonstration of a fusion environment^3–6^. It is thought that He bubbles play an important role in the growth mechanism of the fuzzy structure. Following the initial identification, similar nanostructure growth have been identified on various metals including molybdenum (Mo), rhenium (Re), nickel (Ni), iron (Fe), titanium (Ti), and tantalum (Ta)^7–13^. For nuclear fusion devices, the nanostructure growth could change the plasma material interaction completely, especially with regard to the influence of transients accompanied by plasma instabilities called edge localized modes (ELMs)^14^. Because the material property would be altered significantly^15–18^, transients could easily lead to melting^19,20^ and initiation of unipolar arcing^21,22^; the fuzz layer could also be a protective layer for producing cracks^22^. Industrial applications of the developed nanostructures have been explored as well for solar light absorber^23^ and photocatalysis to split water^24^ and decompose organic material using visible light^12,25^. One issue to be investigated further is the growth rate of nanostructures in various environments. In a fusion reactor, the question of whether the fuzz will actually be formed on plasma facing materials remains unanswered. This is because the necessary conditions shown in^7^, regarding the temperature and the incident ion energy, may not be satisfied in so-called detached or partially detached regimes. In such regimes, the electron temperature is much less than 10 eV around the strike point; however, the effects of ELMs have yet to be fully understood and can enhance the fuzz growth^26^. For industrial application, it has been shown that the same structures can be grown in commercially available magnetron sputtering devices^27–29^. However, the growth rate is so low that it takes quite a long time, generally 5–10 hours, to grow the structure (≤1 *μ*m) with its typical He ion flux of ~10^20^ m^−2^ s^−1^ ^29^.

In this study, we show enhanced growth of large-scale metallic nanostructures by He plasma irradiation with the help of an additional precipitation of metallic particles on the surface. In conventional He plasma irradiation, the nanostructured layer is typically up to ~10 *μ*m thick for 10 hours in linear devices. The enhanced growth shown in this study increased the thickness by two orders of magnitude, typically to 1 mm, in a shorter time period. This study shows that a visible metallic large-scale fuzzy layer covers the metal surface after an hour of irradiation. With systematic irradiation experiments and the observation of the developed material, we show the conditions necessary for the growth of the large-scale nanostructure and discuss the potential mechanism of the enhanced fuzzy metallic nanostructure growth.

## Results

**Figure 1:**
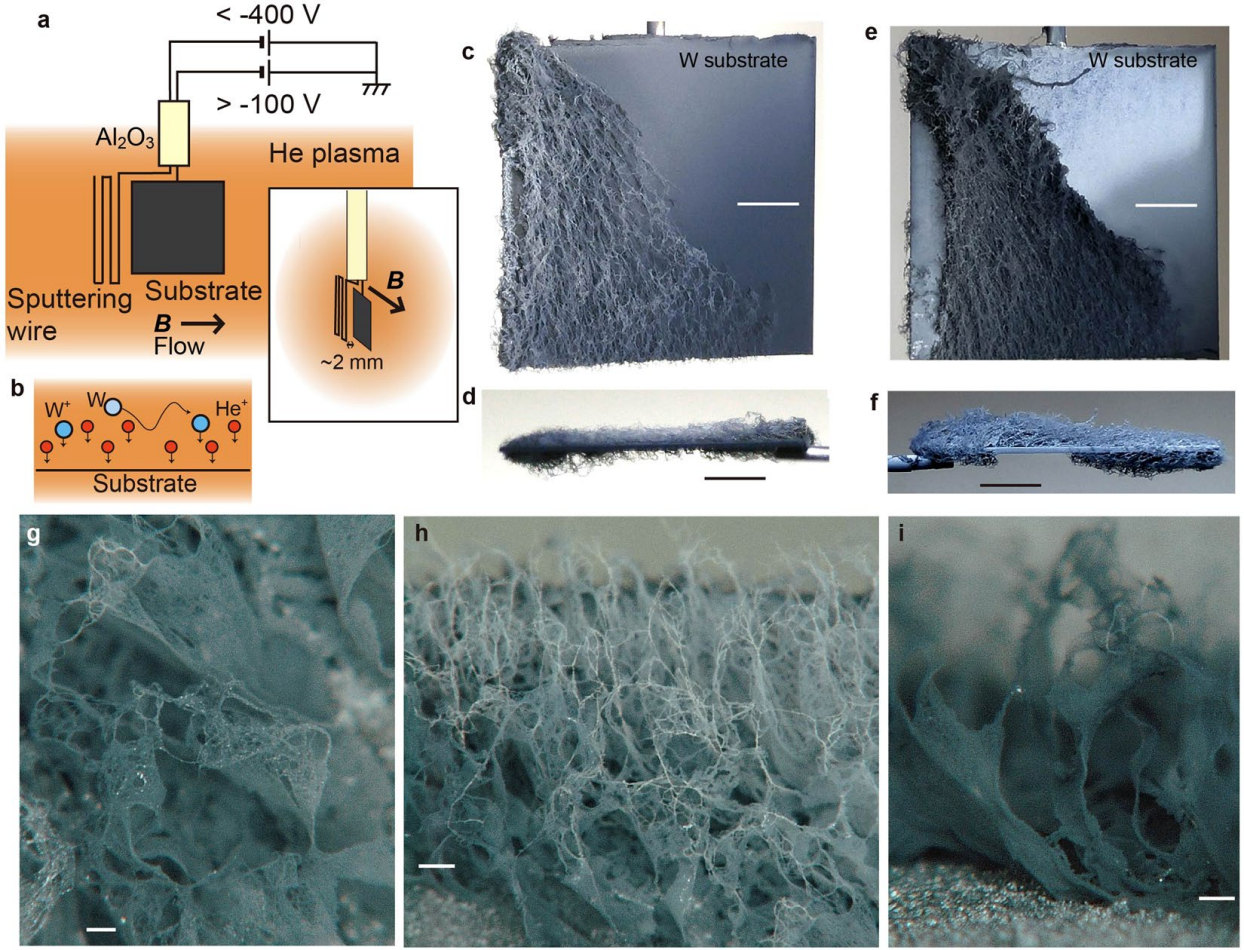
Schematics of the experiments and pictures and micrographs of fuzzy fur like materials. (**a**) Schematic of the experimental setup. (**b**) Schematic of the sample exposed to ions. (**c**,**d**) Pictures of W large-scale fuzz on W substrate for the irradiation time, *t*, of 3600 s from top and side. The sample temperature, *T*_s_, the incident ion energy, *E*_i_, and the ion fluence, Φ, were 1200 K, 70 eV, and 1.8 × 10^25^ m^−2^, respectively. (**e**,**f**) Pictures of Mo large-scale fuzzy nanostructures, and the irradiation conditions were *T*_s_ of 1250 K, *E*_i_ of 70 eV, and Φ of 2.0 × 10^25^ m^−2^. (**g**–**i**) Optical microscope micrographs of W large-scale fuzzy nanostructures. The irradiation conditions were *T*_s_ of 1300 K, *E*_i_ of 70 eV, and Φ of 2.8 × 10^25^ m^−2^. Scale bar in (**c**–**f**) and (**g**–**i**) represents 2 and 0.1 mm, respectively.

Figure 1a shows a schematic of the experimental setup of the plasma device NAGDIS-II (Nagoya Divertor Simulator). In addition to a substrate exposed to the He plasma, a meander shaped W or Mo wire (sputtering wire) was installed close to the sample. The distance between the sputtering wire and the corner of the sample was ~2 mm at the closest location, as shown in Fig. 1a. Since the mean free path of ionization of W atoms from the sputtering wire is comparable or shorter than the distance to the substrate, the substrate would be exposed to W ions in addition to He ions, as shown in Fig. 1b. In this configuration, the sample surface was in parallel with the magnetic field line. A plasma flow exists in the same direction as the magnetic field line in parallel direction and clockwise direction in azimuthal direction due to ***E ***× ***B*** drift as seeing from the upstream direction. The sputtering wire was not on the same magnetic field line, but ~1 mm offset from the sample plane. Figure 1c,d illustrates a W sample after 3600 s irradiation from top and side, respectively. This is different from conventional He plasma irradiation, in which the surface becomes totally black with the formation of the nanostructured layer; rather, the sample surface was covered with visually identifiable fur-like material with a thickness of 0.5–1 mm. The structure was mainly grown on the front, but it also extended slightly to the back as well. To check whether the difference was actually caused by the wire, we moved the wire to the right side and confirmed that the visible fuzzy structures grew from the opposite side of the sample. Also, we have conducted experiment in a configuration in which magnetic field is normal to the sample. However, we focused on the parallel configuration shown in Fig. 1a in this study, since the growth was more clearely observed.

In Fig. 1e,f, the irradiation was conducted in the combination of W substrate and a Mo sputtering wire. It was observed that the W substrate was covered with Mo fuzzy fur-like material. It was noted that the fur-like material was not developed when the substrate was Mo under similar conditions, probably because the temperature range of the nanostructure formation on Mo is less than 1050 K^30^. If the substrate was W, on the other hand, the fuzz would grow from the bottom, and the structure could be the seed to enhanced fuzz growth. We conducted energy-dispersive X-ray spectroscopy (EDS) for grown structures in W and Mo cases; only W and Mo for W and Mo sputtering wire cases, respectively, were identified other than oxygen, which is always detected after the exposure to the air. Although it would be necessary to choose appropriate substrate and irradiation conditions, it would be possible to develop fuzzy fur with various metals. Hereafter, we focused on the case with W sputtering wire and W substrate.

Before detailing the features of the grown structure with microscope analyses, we present a rough mass balance between the sputtering wire and sample and the estimated porosity of the grown structure. We conducted mass measurements of a sample before and after the irradiation, using an electronic scale (BM-22, A&D Co.). For two-hour irradiation, the W sputtering wire, which was biased −500 V, lost 34.80 mg; the W sample gained 1.90 mg, roughly 5% of the sputtered material. Assuming that deposited W has the same density as that of the bulk W, the thickness would be 0.98 *μ*m; the deposition rate was ~500 nm/h, assuming that the deposition occurred on single side of the sample surface (100 mm^2^). During irradiation, the large-scale fuzzy structure covered approximately half the sample surface (50 mm^2^), and the thickness estimated from an image taken from the side was ~0.9 mm. We removed the large-scale fuzzy structure from the sample; its mass totaled 0.91 mg, indicating roughly half the precipitated W contributed to the large-scale fuzzy structure growth. Although it is not easy to estimate the actual volume accurately, assuming that the thickness was uniform, we estimated that the mass density of the large-scale fuzzy structure was roughly 0.02 g/cm^3^; this is three orders of magnitude less than actual W mass density of 19.25 g/cm^3^. Even considering the ambiguity of the estimation, we can say that the grown structure is much more porous than a conventional fuzz structure, the porosity of which is ~95% when the thickness is several *μ*m^31^.

Figure 1g–i shows optical microscope images of the W fur-like material. The images show that the large-scale fuzz comprised fiberform and membrane. The fibers were connected to each other; some parts had mesh-like features with dense fibers. In later images, those fibers and membranes comprised much finer structures.

**Figure 2:**
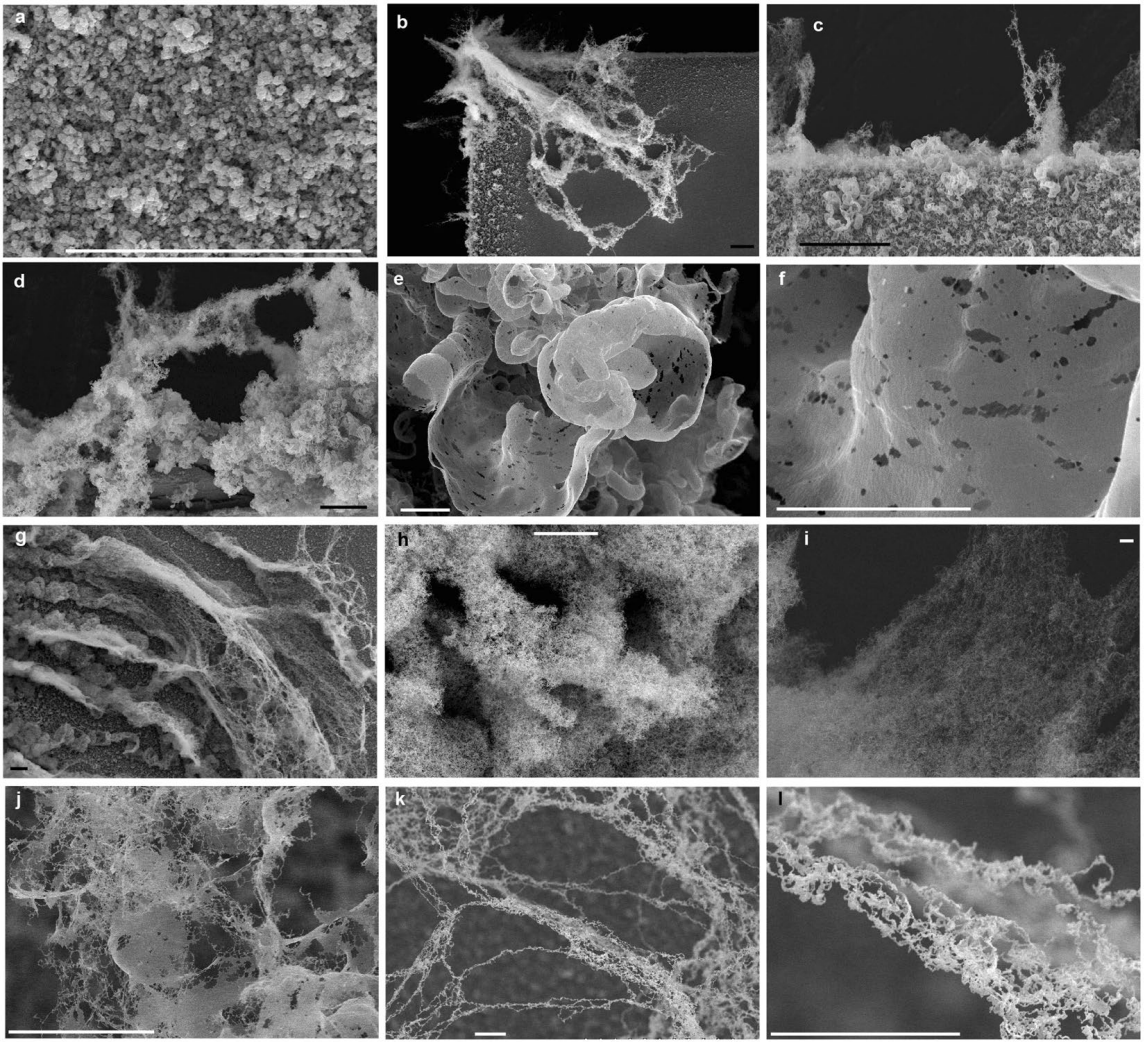
Various microscopic features of large-scale fuzz structures observed by SEM. The sample temperature, *T*_s_, the incident ion energy, *E*_i_, the He ion flux, Γ, to the sample and the exposure time, respectively, were as follows: (**a**,**b**,**c**,**h**,**j**) 1200 K, 70 eV, 4.9 × 10^21^ m^−2^ s^−1^, and 1800 s, (**d**,**e**,**f**) 1200 K, 70 eV, 4.8 × 10^21^ m^−2^ s^−1^, and 3200 s, (**k**,**l**) 1300 K, 70 eV, 7.7 × 10^21^ m^−2^ s^−1^, and 3600 s, and (**k**,**l**) 1300 K, 70 eV, 6.5 × 10^21^ m^−2^ s^−1^, and 3600 s. The position in (**a**) corresponds to several mm from the right edge (non-growth part), and in (**b**–**l**) correspond to fuzzy part of the structures. Scale bars represent (**b**,**c**,**d**,**g**) 100 *μ*m (black bar) and (**a**,**e**,**f**,**h**,**i**,**j**,**k**,**l**) 10 *μ*m (white bar).

A scanning electron microscope (SEM) was used to observe several samples with large-scale W fuzzy structures. The observation location in Fig. 2a corresponds to a part where no fuzzy structures were grown. The surface was rough, probably due to deposition, but no fine fiber or film structures were identified. The rough surface, the typical roughness scale length of which was several hundred nm, was probably formed by the combination of the growth process of protrusions and deposition on the surface. Because of the deposition, the color of the surface was silver; this was rather different from the totally black surface that fuzzy nanostructured surfaces usually have. Figure 2b,c show SEM micrographs of the edge part of a sample, where initial growth can be identified. Fine structures with the height greater than 0.2 mm were grown from the edge. Figure 2d–f show the location where structures grew further. Larger structures were composed of micrometer-sized petal-like structures; the membranes were thin and curled, and had many holes. As shown in Fig. 2e, the membranes were always curled inward. If the He flux on the one side of a membrane was higher than the other side, a curling might have occurred toward lower flux side, because the swelling could be greater on the higher flux side. Especially on top, structures were composed of more fiber-like structures or combinations of membranes and fibers, as shown in Fig. 2g–j. In some parts where large-scale fuzzy structures had grown considerably, fine nanofibers were entangled and grown extensively, as shown in Fig. 2k,l.

**Figure 3:**
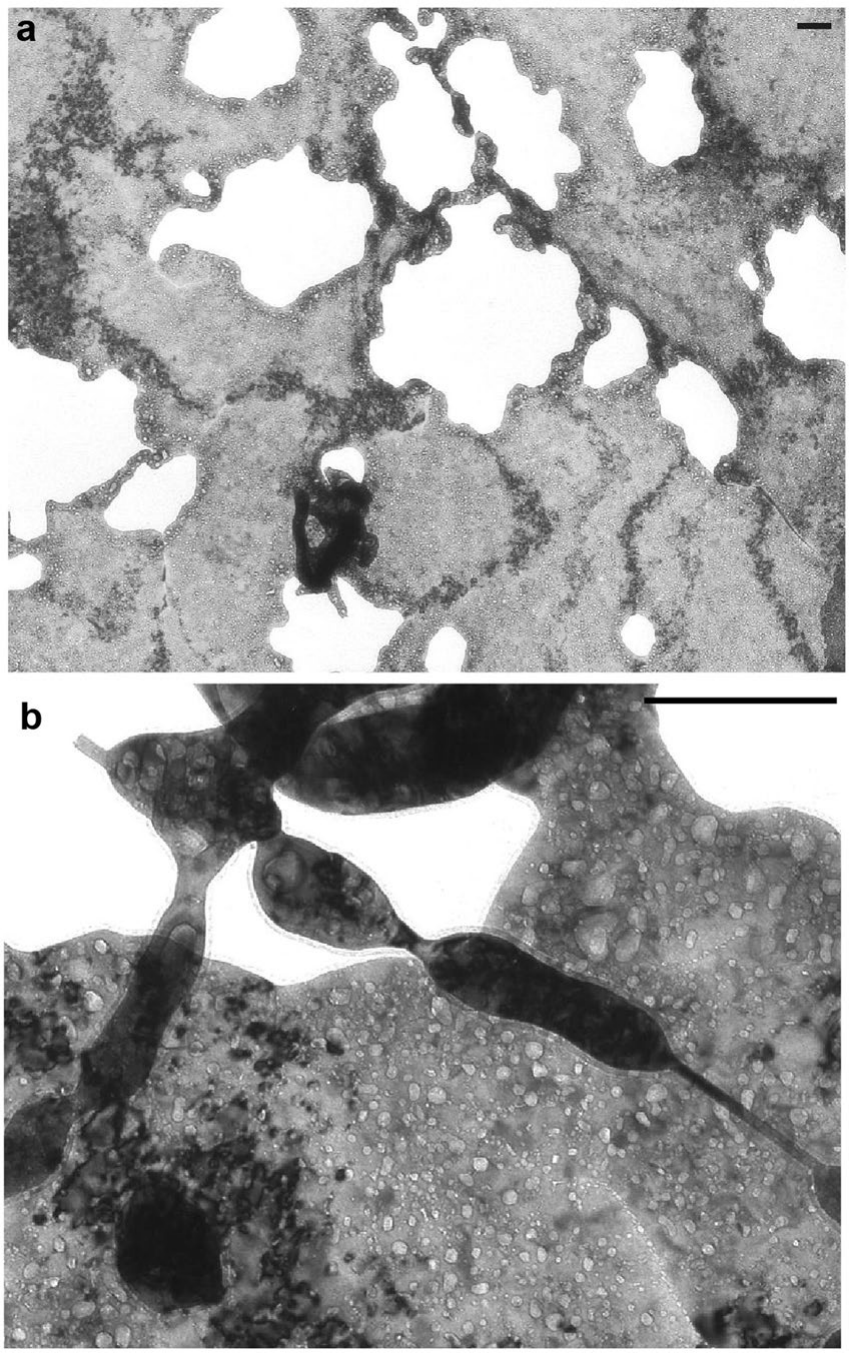
TEM micrographs of the large scale nanostructures. The irradiation conditions are as follows: *T*_s_ of 1200 K, *E*_i_ of 70 eV, the ion flux, Γ, of 4.8 × 10^21^ m^−2^ s^−1^, and *t* of 3200 s. Scale bar represents 100 nm.

Figure 3a,b shows transmission electron microscope (TEM) micrographs of the mixed fiber membrane part of a sample. As seen in Fig. 3a, streaks with darker contrast, which indicates curvature of the membrane (not defections), were identified inside the thin membrane. This suggested that the parts were originally composed of fibers and grown to membrane; TEM micrographs suggested that fiberform structures grew initially and developed network structures, which then formed membranes with the help of deposition on the structure. Figure 3b is an enlarged micrograph focusing on a membrane. Many He bubbles less than 20 nm were grown on the membrane and fiberform structures. Since the brightness of the image should correspond to the thickness under kinematic conditions, one can say that the thickness of the membrane is ~20–30 nm.

**Figure 4:**
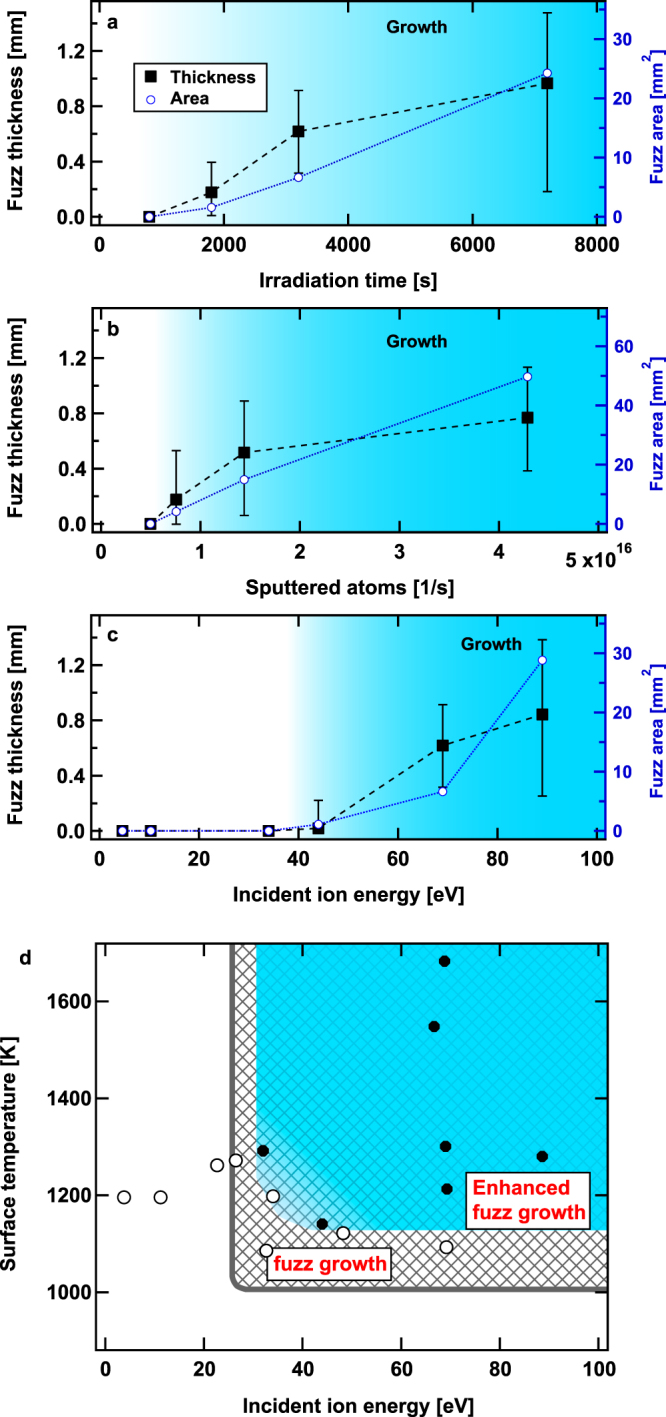
Necessary conditions for the enhanced fuzz growth. The thickness and area of the fuzzy structure as a function of (**a**) the irradiation time, (**b**) the number of sputtered atoms, and (**c**) the incident ion energy. In (**a**,**b**,**c**), the closed markers represent averaged thickness subtracting the original thickness, and the associated error bars are the minimum and maximum values. (**d**) Enhanced fuzz growth conditions in terms of the incident ion energy and the surface temperature. In (**a**) *T*_s_ ~ 1200 K, *E*_i_ = 70 eV, and Γ = 4.8 ± 0.2 × 10^21^ m^−2^ s^−1^. In (**b**) *T*_s_ = 1250−1310 K, *E*_i_ = 70 eV, Γ = 5−7 × 10^21^ m^−2^ s^−1^, and *t* = 3600 s; the potential of the sputtering wire was changed from −290 to −500 V. In (**c**) *T*_s_ = 1140–1200 K, *E*_i_ = 70 eV, Γ = 2 × 10^21^ m^−2^ s^−1^, and *t* = 3200–3600 s. In (**d**) closed and open markers represent cases where large-scale fuzz structure was observed and not observed, respectively, after irradiation.

We investigated the conditions required for the growth of the large-scale fuzz. Figure 4a shows the thickness and area of the large-scale fuzz as a function of irradiation time. The thickness and area were measured from images taken from the side and top, respectively; the original thickness of the sample (0.2 mm) was subtracted to estimate the thickness. We observed that the growth started around 1000 s. After about two hours of irradiation, the thickness grew to 1 mm and approximately 25% of the surface was covered with the large-scale fuzz. When the irradiation time was 2 h (7200 s), about 1/4 of the total area was covered with the fuzzy structure. It is known that an incubation He ion fluence existed before the fuzzy nanostructure growth began^32^. Similarly, the enhanced fuzz growth began after irradiation had continued for some time. The fluence around 1000 s was 5 ×10^24^ m^−2^, which was almost consistent with the incubation He ion fluence previously observed in our device^33^. Figure 4b shows the large-scale fuzz growth as a function of the sputtered atoms. A theoretical sputtering rate was used to deduce the sputtered atoms^34^. We observed a threshold value around 5 × 10^15^ 1/s in the present condition. Although the mass change was not measured for the samples used in Fig. 4b, based on the sputtering rate of the sample, the mass variation of which was measured, it was assessed that the deposition rate was ~40 nm/h at 5 × 10^15^ 1/s.

From Fig. 4b, it is clear that no large-scale fuzz growth occurred without sufficient supply of particles. In other words, if the amount of precipitated particles is less than the threshold, conventional fuzz growth occurs without large-scale fuzz growth. Note that the threshold value should change if the distance between the sputtering wire and the substrate was altered and can be altered when changing the surrounding plasma condition. Figure 4c shows the incident ion energy dependences of the thickness and area covered with large-scale fuzzy structure. The enhanced fuzz growth was identified when the incident ion energy was greater than ~40 eV, and the growth rate, especially the areal growth rate, increased with the incident ion energy. It was suggested that there exists an energy threshold similar to the conventional fuzz growth^7^.

Figure 4d summarizes the necessary temperature and the incident ion energy conditions for conventional fuzz growth and enhanced fuzz growth. The temperature range of 1000–2000 K and the incident ion energy of >20–30 eV were required for conventional fuzz growth^7^. The condition required for enhanced fuzz growth was well overlapped and within the conventional fuzz growth condition, and the minimum temperature and energy seemed to be slightly, i.e., ~100 K and 10 eV, respectively, higher. The differences in the minimum energy and temperature boundaries between the conventional fuzz and large-scale fuzz were subtle. The temperature at the top of the large-scale fuzz might have been different from the bulk, and the large-scale fuzz could be formed when we increased the irradiation time; the boundaries may be slightly altered with further experiments. In this study, we could not identify the maximum temperature range, because significant electron emission was initiated from the sputtering wire in the high density range.

## Discussion

Concerning the conventional fuzz growth, the mechanism of the growth process was investigated experimentally^5,7^, theoretically^35,36^, and numerically^37–39^. Since the growth rate saturated and did not increase with the flux if it was greater than 10^22^ m^−2^ s^−1^, the average growth rate was roughly 1 × 10^−3^ *μ*m/s for 1 *μ*m thick at 1120 K^32^. The growth rate decreased with the irradiation time; a time on the order of 10^8^ s, i.e., three years, was required to grow a 1 mm fuzzy layer when we extrapolated from the conventional growth model. In this study, under metallic ion precipitation conditions, the growth rate was 100 times greater for a 1 *μ*m thick layer and five orders of magnitude greater for a 1 mm thick layer compared with the conventional value.

**Figure 5:**
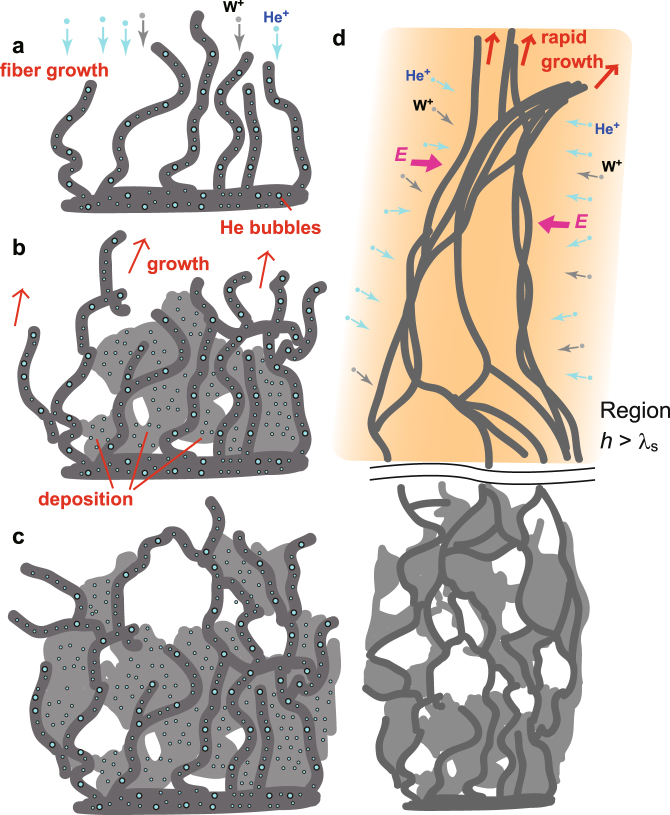
Schematics of the enhanced fuzzy nanostructure growth process. (**a**,**b**,**c**) Schematics of initial enhanced growth processes with the formation of fibers and growth of membrane with deposition. (**d**) Schematic of further enhanced growth process when the structure size *h* is greater than sheath thickness *λ*_s_. The electric field formed around the fibers started collect ions to thin fibers. These schematics are drawn based on the TEM and SEM micrographs in Figs 2k and 3a.

Although the growth process has yet to be fully understood, here we discuss the potential processes related to enhanced fuzz growth using the schematics in Fig. 5. The growth of fiberform structure was accompanied by the He bubble growth^33^. With additional deposition of metallic ions, fibers grew to membranes. Membrane structures were dominated on the surface in the initial growth phase, as seen in Fig. 2c,d. The fraction of membrane structure seemed to be higher at the position close to the bulk surface. Because sufficient adatoms could be formed on the fibers, the growth rate would not decrease even if the fiberform structures were grown. The fact that the fuzzy layer thickness was proportional to the square root of irradiation time suggested that some diffusion process controlled the growth rate of nanostructures for conventional fuzz growth^5,40^. From simulations and theoretical investigation, it has been discussed that the growth rate may be explained in terms of adatom diffusion over fibers^36^, formation of bubbles and growth of loop punching^37^, and ballistic penetration of He atoms^41^. Significant increase of the growth rate of nanostructures indicated that the growth process was not controlled by the same diffusion process when precipitation of metallic ions was introduced. Furthermore, concerning the formation of long fiber structures shown in Fig. 2k,l, another process should be introduced. If the structure’s height, *h*, was much smaller than the sheath thickness, *λ*_s_, the thickness being roughly 100 *μ*m under the present condition^42^, the grown structure would hardly influence the trajectory of ions. The orbit of ions could be modified by structures in the sheath even if the structural size was less than the sheath thickness. A ballistic simulation is required to investigate whether ions can be captured by fiberform structures when *h* < *λ*_*e*_, because ions have already been accelerated in the normal direction to the surface by the electric field in sheath and pre-sheath. However, when *h* was comparable to or greater than *λ*_s_, the sheath structure would change from flat to the structure influenced by the grown structures, and, consequently, the electric field formed around the fine structures should start to capture ions. Even if the width of the fiberform structure is 10–20 nm, ions captured by the electric field would accelerate the growth of the fiberform structures since the Debye length is on the order of 10 *μ*m. In other words, the width of fibers would be significantly increased electrostatically, because sheath could be started to be formed around the fibers and ions would be accelerated toward fibers; this is shown in Fig. 5d. When we observe the large scale nanostructures from top, fiberform structure or mixed fiber-membrane structures covered the top parts, while the petal-like structures and membrane structures dominated on the bottom part. It was likely that the structures grown in the enhanced growth process described in Fig. 5d formed long fibers, and further deposition changed them to combination of fibers and membranes.

The growth was always originated from the left side of samples in the present configuration. Before the fur-like structures were expanded on the surface, nanostructures with 0.1 mm in height, which are comparable to the sheath thickness, were often observed on the left edge of the sample, as seen in Fig. 2c. It was likely that fur-like materials were grown from those structures. If such large structures were extended from the surface, an enhanced growth, shown in Fig. 5d, could have been triggered. As seen in Fig. 1c and e, the structures were always grown in downward direction from the edge close to the sputtering wire. The direction was same as ***E ***× ***B*** direction for the sheath electric field formed around the sample and the direction of the ***E ***× ***B*** plasma rotation. If the electric field around the sample was influential, the magnetic field effects on the sheath and pre-sheath should be taken into account to understand the trajectory of ions to the surface. The particle in cell (PIC) simulation will be helpful to understand the appropriate sheath structure around the sample considering elastic and Coulomb collisions, plasma flow, and so on.

One of the mysteries of these processes is why the structural width/thickness always remains at a scale of tens of nanometers and does not grow further, even with additional metallic particle precipitation. It is likely that adatoms formed by ion precipitation contribute to the growth in height primarily due to surface diffusion, adatom migration, and/or swelling of structures. As with conventional fuzz, the fine structures would be retained with the help of He irradiation and reintegrated to the surface when the structures were heated up without He irradiation^43^. Also, TEM suggested that deposition of precipitated metallic atoms filled in the region between fibers and grew to membrane. However, the thickness of the membranes still had the same width of the nanofibers. Further experimental and theoretical/numerical works will be required to understand the mechanisms of the growth. Those investigations may help to fully understand the growth of conventional fuzz growth process.

Recently, nano-tendril bundles (NTBs) have been observed in MIT’s group when the incident He ion energy was modulated^44^. In NAGDIS-II, NTBs have also been identified when background pressure was not sufficiently low and some amount of sputtering from the sample occurred^45^. The height of the nano-tendril bundles could be greater than 50 *μ*m, in which case it is unlikely that the growth rate is conventional. In fusion devices, gases such as argon, neon, or nitrogen are injected to control the heat load to the divertor by enhancing radiation. Thus, in addition to incident ion energy modulation, the sputtered atoms from the surface can play a significant role in nanostructure growth. It would be interesting to investigate whether the enhanced fuzz growth process and/or formation of NTBs is triggered in fusion devices in response to ELMs, which would convey higher energy ions to lead to considerable sputtering. On the other hand, He plasma irradiation with additional metallic ions can be used for mass production of metallic fuzz for industrial applications. Various combinations of sputtering wire and substrate materials are of interests to be investigated in the future.

## Methods

### The linear plasma device NAGDIS-II

Experiments were conducted in the linear plasma device NAGDIS-II^46^. He plasmas were formed by a direct current arc discharge using a heated LaB_6_ cathode plate heated with a carbon heater. The magnetic field, the strength of which was typically <0.25 T, formed column-shaped plasma with a length of ~2.5 m. In the NAGDIS-II device, high density low temperature plasmas can be produced in a steady state; the typical density and temperature ranges were, respectively, 10^18^–10^19^ m^−3^ and 0.1–0 eV. In this study, the magnetic field strength was in the range of 0.05–0.175 T.

### He plasma irradiation

Tungsten samples (10 × 10 × 0.2 mm^3^) were installed and exposed to He plasma ~1.5 m downstream from the plasma source in the NAGDIS-II device. The incident He ion energy was controlled by negatively biasing the sample against the space potential of the plasma, which is ~−5 V. The surface temperature was increased to above 1000 K by the plasma bombardment without any additional heating. The surface temperature was measured by a radiation thermometer; the irradiation was started before switching on the sputtering wire biasing, because the radiation from the sputtering wire would disturb the measurement. W and Mo wire with a diameter of 0.5 mm was used for the sputtering wire. The sputtering wire was biased negatively deeper (<−250 V) so that significant sputtering would occur, although the incident ion energy to the substrate was less than 100 eV, where the sputtering by He^+^ rarely occurs. In the case of W, the mean free path of W atoms from the sputtering wire was 1.7 mm for a typical density of 2 × 10^18^ m^−3^, considering the ionization rate coefficient of 8 × 10^−13^ m^3^/s at 5 eV^47^ and the mean energy of 7 eV^34^; many of the sputtered atoms would be ionized before reaching the surface.

### SEM and TEM observation

After the irradiation experiments, some samples were analyzed by SEM and TEM. For TEM, the fragments of the large-scale fuzz were peeled off from the substrate, sandwiched with micro grids with carbon films (#10-1012 Elastic Carbon Film ELS-C10, Okenshoji Co., Ltd.) and observed.
